# Matrix metalloproteinases in the mouse retina: a comparative study of expression patterns and MMP antibodies

**DOI:** 10.1186/s12886-015-0176-y

**Published:** 2015-12-29

**Authors:** Lies De Groef, Lien Andries, Kim Lemmens, Inge Van Hove, Lieve Moons

**Affiliations:** Laboratory of Neural Circuit Development and Regeneration, Animal Physiology and Neurobiology Section, Department of Biology; KU Leuven, Naamsestraat 61, Box 2464, B-3000 Leuven, Belgium

**Keywords:** Matrix metalloproteinase, Retina, Immunohistochemistry, Western blot, Antibodies

## Abstract

**Background:**

Matrix metalloproteinases (MMPs), a family of Zn^2+^-dependent endoproteases, have been shown to act as fine regulators of both health and disease. Limited research revealed that they are essential to maintaining ocular physiology and inordinate MMP activities have been linked to several neurodegenerative disorders of the retina, including age-related macular degeneration, proliferative diabetic retinopathy and glaucomatous optic neuropathies (GONs). Nevertheless, a clear definition of their pathology-exacerbating and/or -resolving actions is lacking, especially in the context of GONs, as most studies thus far merely focused on expression profiling in human patients. Therefore, in an initial step towards an improved understanding of MMP functions in the retina, we studied the spatial expression pattern of MMP-2, -3, -9 and MT1-MMP in the healthy mouse retina.

**Methods:**

The spatial expression pattern of MMP-2, -3, -9 and MT1-MMP was studied in the healthy mouse retina via immunohistochemical stainings, and immunoreactivity profiles were compared to existing literature. Moreover, we considered sensitivity and specificity issues with commercially available MMP antibodies via Western blot.

**Results:**

Basal expression of MMP-2,-3, -9 and MT1-MMP was found in the retina of healthy, adult mice. MMP-2 expression was seen in Müller glia, predominantly in their end feet, which is in line with available literature. MMP-3 expression was described for the first time in the retina, and was observed in vesicle-like structures along the radial fibers of Müller glia. MMP-9 expression, about which still discords exists, was seen in microglia and in a sparse subset of (apoptosing) RGCs. MT1-MMP localization was for the first time studied in adult mice and was found in RGC axons and Müller glia, mimicking the MT1-MMP expression pattern seen in rabbits and neonatal mice. Moreover, one antibody was selected for each MMP, based on its staining pattern in Western blot.

**Conclusions:**

The present MMP immunoreactivity profiles in the mouse retina and validation of MMP antibodies, can be instrumental to study MMP expression in mouse models of ocular pathologies and to compare these expression profiles to observations from clinical studies, which would be a first step in the disentanglement of the exact role MMPs in ocular/retinal diseases.

## Background

Matrix metalloproteinases (MMPs), a family of Zn^2+^-dependent proteases, were originally named after their ability to cleave and remodel the extracellular matrix (ECM), however, their substrate repertoire has proven to be much broader, comprising other proteinases, growth factors, signaling molecules, cell surface receptors, and even intracellular targets. By proteolytic cleavage, MMPs modify the structure and activity of these substrates, and add a complex extra dimension of biological control. As a result, MMPs and their major endogenous inhibitors ‘tissue inhibitors of metalloproteinases’ (TIMPs) are important regulatory nodes in the protease web, a complex network of interactions that regulates protease activities and determines the functional state of the proteome and cell activity [1–3]. Deregulated MMP activity is a common characteristic of many diseases, including neurodegenerative disorders such as glaucoma, multiple sclerosis, Huntington’s disease, Alzheimer’s disease, Parkinson’s disease, *etc.* [4–11]. Nevertheless, despite their detrimental impact during central nervous system (CNS) pathology, there is ample evidence corroborating MMPs as fine regulators of CNS physiology, and well-balanced MMP activity is instrumental to CNS development, plasticity and repair [12–15].

Also in the retina, MMPs, expressed by resident cells, invading vasculature and inflammatory cells, have been associated with pathologies that involve matrix degradation, cell proliferation, neovascularization and inflammation. Briefly, altered MMP activity, often linked to a disturbed MMP/TIMP ratio, has been observed in cases of age-related macular degeneration, proliferative diabetic retinopathy, glaucomatous optic neuropathies (GONs), *etc.* [5, 16, 17]. In proliferative retinopathies, such as age-related macular degeneration and diabetic retinopathy, the primary role of MMPs is believed to be confined to neovascularization, *i.e.* vessel invasion and disruption of the blood-retinal barrier, which ultimately result in retinal degeneration [18–21]. On the other hand, the causal role of MMPs during the pathogenesis of GONs is not yet well-understood, albeit a contribution of MMP-9 to ECM remodeling leading to detachment-induced retinal ganglion cell (RGC) death has been put forward [22–24]. Nevertheless, multiple studies in GON patients and in animal models of spontaneous and experimentally induced GONs, have linked altered MMP expression/activity to GON onset and disease progression. In addition, patient studies have pointed out several polymorphisms in MMP genes as important risk factors for developing GON [25–27].

Altogether, these data have led to the hypothesis that MMPs might be involved in GON pathogenesis. However, the majority of investigations has been conducted in human patients, and limited their focus to quantitative changes in MMP expression and activity levels, rather than determining the spatial localization and function of these proteinases. In addition, interpretation of the available data is compromised by the many contradictions in the multitude of studies in GON patients and animal models. This has several reasons, including insufficient specificity of techniques for localization and quantification of MMP expression/activity, extensive regulation of MMPs at transcriptional, translational and post-translational level, low samples sizes and interindividual variability in MMP expression [28] and the limitations of *in vitro* and *in vivo* animal models. In order to better understand the functional relevance of altered MMP expression and the exact involvement of these endopeptidases in GON, mechanistic studies are needed to interpret expression data obtained in GON patients. A first requisite for the disentanglement of the role of MMPs in GONs, would be to assess whether MMP expression patterns in animals correspond to what has been observed in humans. Therefore, in this manuscript, we describe the spatial expression pattern of MMP-2, -3, -9 and MT1-MMP in the healthy, adult mouse retina, by means of immunohistochemical stainings with different MMP antibodies, combined with double labeling for specific retinal cell markers. In order to consider sensitivity and specificity issues with MMP antibodies, two commercially available antibodies for each MMP were evaluated via Western blot.

## Methods

### Experimental animals

All studies were conducted in compliance with the European Communities Council Directive of 22 September 2010 (2010/63/EU) and the Belgian legislation (KB of 29 May 2013), and were approved by the KU Leuven institutional ethical committee. Adult (2–3 months) wild type mice (C57Bl6) were obtained from the university breeding colony. Animals were kept under a 12/12 h light–dark cycle and had *ad libitum* access to food and water.

### Immunohistochemistry on fixed cryosections

Mice were deeply anaesthetized (30 mg/kg sodium pentobarbital) (Nembutal, Ceva Santé Animale, Libourne, France) and perfused transcardially with 4 % phosphate-buffered paraformaldehyde (PFA) and eyes were dissected. Eyes were postfixed overnight in 4 % PFA, the cornea and lens were removed, and the remainder posterior segment of the eye was cryoprotected in a 10 %-20 %-30 % sucrose series (in 10 mM phosphate-buffered saline (PBS)) and embedded in Tissue-Tek optimal cutting temperature medium (Sakura Finetek, Alphen aan den Rijn, The Netherlands) to make transverse retinal cryosections (10 μm).

For immunostaining of MMPs on these cryosections, antigen retrieval was performed by heating the sections in citrate buffer (10 mM [pH 6.0]) for 20 minutes at 95C, followed by a 20 minutes cool-down. In case tyramid signal amplification was used, sections were incubated for 20 minutes in 0.3 % hydrogen peroxidase (in methanol) to saturate endogenous peroxidases. Next, sections were subjected to a 1 hour blocking step with 20 % pre-immune serum (Life Technologies, Carlsbad, CA), followed by overnight incubation with the primary antibody at room temperature (Table 1). Secondary IgG antibodies were conjugated to biotin (1:300) (Dako, Glostrup, Denmark) and applied for 45 minutes, followed by 30 minutes incubation with streptavidin-horse radish peroxidase (HRP) (1:100) (Perkin-Elmer, Waltham, MA). Finally, fluorescein isothiocyanate (FITC) tyramid signal amplification was performed according to the manufacturer’s instructions (Perkin-Elmer). By exception, for immunostaining with the MT1-MMP antibody RP-3, secondary IgG antibodies were HRP-conjugated (1:300) (Dako) and applied for 45 minutes, followed by FITC tyramid signal amplification. Sections were rinsed with 10 mM Tris-buffered saline (TBS) in between steps, and pre-immune goat serum and antibodies were diluted in 0.5 % blocking solution (Perkin-Elmer) in TBS. 4′,6-diamidino-2-phenylindole (DAPI; 1 μg/ml in PBS) (Applichem, Darmstadt, Germany) was used as a fluorescent nuclear counterstaining and sections were mounted using mowiol anti-fading medium (10 % mowiol 4–88 (Sigma-Aldrich, St. Louis, MO), 40 % glycerol, 0.1 % 1,4-diazabicyclo-[2,2,2]-octane in 0.2 M Tris–HCl [pH 8.5]).

**Table 1:** Experimental details for immunohistochemical (double) stainings for MMPs/TIMP and retinal cell markers on retinal cryosections and retinal flatmounts

	Primary antibody (dilution)	Primary antibody supplier	Secondary antibody (dilution)
Immunohistochemical staining for MMPs on retinal cryosections			
MMP-2	^a^sc-8835-R (1:200) (ab1)	Santa Cruz, Dallas, TX	GAR-B (1:300)
	ab19167 (1:200) (ab2)	Millipore, Billerica, MA	GAR-B (1:300)
MMP-3	ab52195 (1:200) (ab3)	Abcam, Cambridge, United Kingdom	GAR-B (1:100)
	sc-6839-R (1:50) (ab4)	Santa Cruz, Dallas, TX	GAR-B (1:300)
MMP-9	ab58803 (1:100) (ab5)	Abcam, Cambridge, United Kingdom	GAM-B (1:300)
	ab38898 (1:500) (ab6)	Abcam, Cambridge, United Kingdom	GAR-B (1:300)
MT1-MMP	ab53712 (1:200) (ab7)	Abcam, Cambridge, United Kingdom	GAR-B (1:300)
	RP-3 (1:200) (ab8)	Triple Point, Forest Grove, OR	GAR-HRP (1:300)
TIMP-1	^a^ sc-5538-R (1:100)	Santa Cruz, Dallas, TX	GAR-B (1:300)
Double staining for MMPs and retinal cell markers on retinal cryosections			
MMP-2	ab19167 (1:200)	Millipore, Billerica, MA	GAR-HRP (1:300)
+ GS	MAB302 (1:500)	Millipore, Billerica, MA	GAM-Alexa (1:200)
MMP-3	sc-6839-R (1:50)	Santa Cruz, Dallas, TX	GAR-HRP (1:300)
+ Brn3a	MAB1585 (1:100)	Millipore, Billerica, MA	GAM-HRP (1:300)
MMP-3	ab52195 (1:200)	Abcam, Cambridge, United Kingdom	GAR-HRP (1:300)
+ GS	MAB302 (1:500)	Millipore, Billerica, MA	GAM-Alexa (1:200)
MMP-9	ab58803 (1:100)	Abcam, Cambridge, United Kingdom	GAM-B (1:300)
+ Iba-1	019-19741 (1:1000)	Wako, Osaka, Japan	GAR-Alexa (1:200)
MT1-MMP	ab53712 (1:200)	Abcam, Cambridge, United Kingdom	GAR-HRP (1:300)
+ GS	MAB302 (1:500)	Millipore, Billerica, MA	GAM-Alexa (1:200)
MT1-MMP	RP-3 (1:200)	Triple Point, Forest Grove, OR	GAR-HRP (1:300)
+ GS	MAB302 (1:500)	Millipore, Billerica, MA	GAM-Alexa (1:200)
(Double) staining for MMPs and/or retinal cell markers on retinal flatmounts			
MMP-9	ab38898 (1:500)	Abcam, Cambridge, United Kingdom	DAR-B (1:300)
+ Brn3a	sc-31984 (1:750)	Santa Cruz, Dallas, TX	DAG-Alexa (1:200)
MT1-MMP	ab53712 (1:200)	Abcam, Cambridge, United Kingdom	GAR-B (1:300)
+ RT-97	RT-97 (1:200)	Developmental Studies Hybridoma Bank, University of Iowa	GAM-Alexa (1:200)
MMP-2	sc-8835-R (1:200)	Santa Cruz, Dallas, TX	GAR-Alexa (1:500)
GFAP	Z0334 (1:2000)	Dako, Glostrup, Denmark	GAR-Alexa (1:500)

*GAR* goat anti-rabbit IgG, *GAM* goat anti-mouse IgG, *DAR* donkey anti-rabbit IgG, *DAG* donkey anti-goat IgG, *−Alexa* Alexa fluorophore-conjugated, *−B* biotin-conjugated, *GS* glutamine synthetase

^a^ immunostaining on unfixed cryosections

For double immunostainings on cryosections, sections were treated as described above and primary antibodies were simultaneously applied overnight (Table 1). The second day, the Alexa 594-conjugated secondary IgG antibody (1:200) (Life Technologies) was applied together with the HRP-conjugated secondary IgG antibody (1:300) for 1 hour, and the FITC tyramid signal amplification protocol was continued. Alternatively, the Alexa 594-conjugated secondary IgG antibody (1:200) was applied together with the biotin-conjugated secondary IgG antibody (1:300) for 1 hour, followed by 30 minutes incubation with streptavidin-HRP (1:100) and FITC tyramid signal amplification according to the manufacturer’s instructions. For the double staining of MMP-3 and Brn3a, by exception, sections were incubated for 45 minutes with HRP-conjugated anti-rabbit IgG (1:300) followed by FITC tyramid signal amplification. Next, after 3 times 5 minutes rinsing with PBS, sections were incubated for 45 minutes with biotin-conjugated anti-mouse IgG (1:300), followed by 30 minutes incubation with streptavidin-HRP (1:100) and cyanine-3 tyramid signal amplification according to the manufacturer’s instructions (Perkin-Elmer). 4′,6-diamidino-2-phenylindole (DAPI; 1 μg/ml in PBS) was used as a fluorescent nuclear counterstaining and sections were mounted using mowiol anti-fading medium.

### Immunohistochemistry on unfixed cryosections

Mice were deeply anaesthetized (30 mg/kg sodium pentobarbital) and sacrificed by cervical dislocation. Eyes were dissected, rinsed in PBS and immediately embedded in Tissue-Tek optimal cutting temperature medium to make transverse retinal cryosections (10 μm).

Prior to immunostaining of MMP-2 or TIMP-1, with antibody sc-8835-R or sc-5538-R respectively (Table 1), unfixed sections were post-fixed for 10 minutes with 4 % PFA. Next, immunostaining was performed as described above, with omission of the antigen retrieval step.

### Immunohistochemistry on retinal flatmounts

Mice were deeply anaesthetized (30 mg/kg sodium pentobarbital) and sacrificed by cervical dislocation. Eyes were fixed for 1 hour in 4 % PFA, retinas were dissected and flatmounted and again fixed for 1 hour in 4 % PFA. Prior to immunohistochemistry, retinal flatmounts were rinsed in PBS with 0.5 % Triton X-100 for 3 times 10 minutes.

For immunostainings on retinal flatmounts, retinas were frozen for 15 minutes at −80C, before applying the primary antibod(y)(ies) (Table 1). The next day, for single stainings, an Alexa-conjugated secondary IgG antibody (1:500) was applied for 2 hours. For double stainings, the Alexa 594-conjugated secondary IgG antibody (1:200) was applied together with the biotin-conjugated IgG antibody (1:300) for 1 hour, followed by 30 minutes incubation with streptavidin-HRP (1:100) and FITC tyramid signal amplification. Retinal flatmounts were rinsed with PBS with 0.5 % Triton X-100 in between steps, and all antibodies were diluted in PBS containing 2 % Triton X-100 and 2 % pre-immune goat or donkey serum (Life Technologies). Retinal flatmounts were mounted using mowiol anti-fading medium.

Antibodies, dilutions and experimental details are summarized in Table 1. For all stainings, negative controls were included by omitting the primary antibody and by replacing the primary antibody with IgG isotype control antibodies (Life Technologies) in the same dilution. All images were taken with an inverted confocal microscope (FV1000, Olympus, Tokyo, Japan) and processed using FluoViewer 4.0 (Olympus) and Photoshop CS5 (Adobe, San Jose, CA) software. Figures show representative images of at least 3 technical repeats with samples from minimum 3 mice.

### Western blot

Mice were deeply anaesthetized (30 mg/kg sodium pentobarbital) and sacrificed by cervical dislocation. Retinas were quickly dissected and homogenized in ice-cold lysis buffer (50 mM Tris–HCl [pH 7.6], 10 mM CaCl_2_, 150 mM NaCl, 0.05 % Brij-35 (Sigma-Aldrich), 1 % Triton X-100, 100 μM phenylmethylsulfonylfluoride), supplemented with EDTA-free proteinase inhibitor cocktail (Roche, Basel, Switzerland). For MT1-MMP, a lysis buffer dedicated to membrane proteins was used, containing 65 mM Tris–HCl and 2 % SDS, supplemented with EDTA-free proteinase inhibitor cocktail (Roche). For each sample, one retina was homogenized in 100 μl lysis buffer. After homogenization, samples were centrifuged, supernatant was collected and protein concentrations were measured using Qubit fluorometric quantitation (Life Technologies).

Retinal homogenates were loaded on 4–12 % SDS-PAGE and transferred onto a polyvinylidine fluoride or nitrocellulose membrane (BioRad, Hercules, CA). After 2 hours of blocking with 5 % Amersham Enhanced Chemiluminescence Blocking Agent (GE Healthcare, Buckinghamshire, United Kingdom) in TBS, membranes were incubated overnight at room temperature with the primary antibodies (Table 2). The next day, membranes were incubated for 45 minutes with HRP-labeled secondary antibody (1:25000) and protein bands were visualized using a luminol-based enhanced chemiluminescence kit (Thermo Scientific, Waltham, MA). Blots were rinsed in TBS in between steps and all antibodies were diluted in 5 % Amersham Enhanced Chemiluminescence Blocking Agent in TBS. Coomassie Blue total protein stain was performed to confirm equal sample loading, and densitometric analysis of the protein bands was performed using Image Lab software (BioRad).

**Table 2:** Protocol and experimental details for Western blotting with MMP and TIMP-1 antibodies on retinal homogenates

	Primary antibody (dilution)	Secondary antibody	Blotting membrane	Amount of protein loaded
MMP-2	sc-8835-R (1:100) (ab1)	GAR-HRP	nitrocellulose	20 μg
	ab19167 (1:200) (ab2)	GAR-HRP	nitrocellulose	
MMP-3	ab52195 (1:200) (ab3)	GAR-HRP	nitrocellulose	20 μg
	sc-6839-R (1:100) (ab4)	GAR-HRP	nitrocellulose	
MMP-9	ab58803 (1:500) (ab5)	GAM-HRP	nitrocellulose PVDF	30 μg
	ab38898 (1:5000) (ab6)	GAR-HRP		
MT1-MMP	ab53712 (1:1000) (ab7)	GAR-HRP	PVDF	50 μg
	RP-3 (1:5000) (ab8)	GAR-HRP	PVDF	
TIMP-1	sc-5538 (Santa Cruz)	GAR-HRP	nitrocellulose	15 μg

*GAR* goat anti-rabbit IgG, *GAM* goat anti-mouse IgG, *PVDF* polyvinylidine fluoride

Antibodies, dilutions and experimental details are summarized in Table 2. Recombinant human (rh) MMP-2 (125 ng) (co-delivered with Amersham MMP-2 Biotrak Activity Assay, GE Healthcare), rhMMP-3 (0.01 ng) (ab39306, Abcam, Cambridge, United Kingdom) and rhMMP-9 (20 ng) (ab39308, Abcam) were loaded as positive controls. All images were taken with the ChemiDoc MP Imaging System (BioRad) and processed using Image Lab (BioRad) and Photoshop CS5 (Adobe, San Jose, CA) software. Figures show representative images of at least 3 technical repeats with samples from minimum 3 mice.

### Gelatin zymography

Samples were prepared as described above for Western blot. Aliquots containing 120 μg of proteins were incubated with 50 μl gelatin-conjugated sepharose beads (gelatin sepharose 4B, GE Healthcare) in equilibrating buffer (0.5 M NaCl, 10 mM CaCl_2_, 0.01 % Tween-20 in TBS) for 20 minutes at room temperature, for affinity precipitation. Next, the beads were rinsed twice with TBS containing 0.5 M NaCl, 10 mM CaCl_2_ and 0.05 % Tween-20, and once with TBS containing 10 mM CaCl_2_ and 0.05 % Tween-20. Finally, gelatinases were eluted with 20 μl zymogram loading buffer (Novex Tris Glycine SDS Sample Buffer, Life Technologies) and loaded on a 10 % gelatin gel (Life Technologies) for electrophoresis. Gels were incubated in 2.5 % Triton-X (in water) for 30 minutes and developed for 2 days at 37C in TBS containing 10 mM CaCl_2_ and 1.25 % Triton X-100. After staining with Coomassie blue (0.5 % in a mixture of 9:9:2 methanol, water and acetic acid) for 3 hours, gels were destained for 2 hours (in a mixture of 9:9:2 ethanol, water and acetic acid) and imaged.

Culture medium from HT1080 human firbosarcoma cells (5 μl), stimulated with microplasmin to activate secreted pro-MMP-2 and pro-MMP-9, was loaded as a control (kind gift from Dr. B. Jonckx). Images were taken with the ChemiDoc MP Imaging System (BioRad) and processed using Image Lab (BioRad) and Photoshop CS5 (Adobe, San Jose, CA) software. Figures show representative images of at least 3 technical repeats with samples from minimum 3 mice.

## Results

Immunostaining on mouse retinal cryosections revealed that all antibodies tested were suited for immunohistochemistry. Moreover, none of the negative controls revealed any artefacts, autofluorescence or nonspecific staining.

### MMP-2 expression in the healthy, adult mouse retina

In order to investigate the expression and localization of MMP-2, or gelatinase A, two polyclonal rabbit anti-MMP-2 antibodies were used. According to the manufacturer, antibody sc-8835-R (Santa Cruz) (referred to as ab1) recognizes epitopes near the C-terminus of human MMP-2, *i.e.* the hemopexin domain. Antibody AB19167 (Millipore) (referred to as ab2) was generated by immunization with a synthetic peptide from the second half of human MMP-2, and therefore recognizes part of the hemopexin domain, hinge region or (part of) the catalytic domain.

**Fig. 1:**
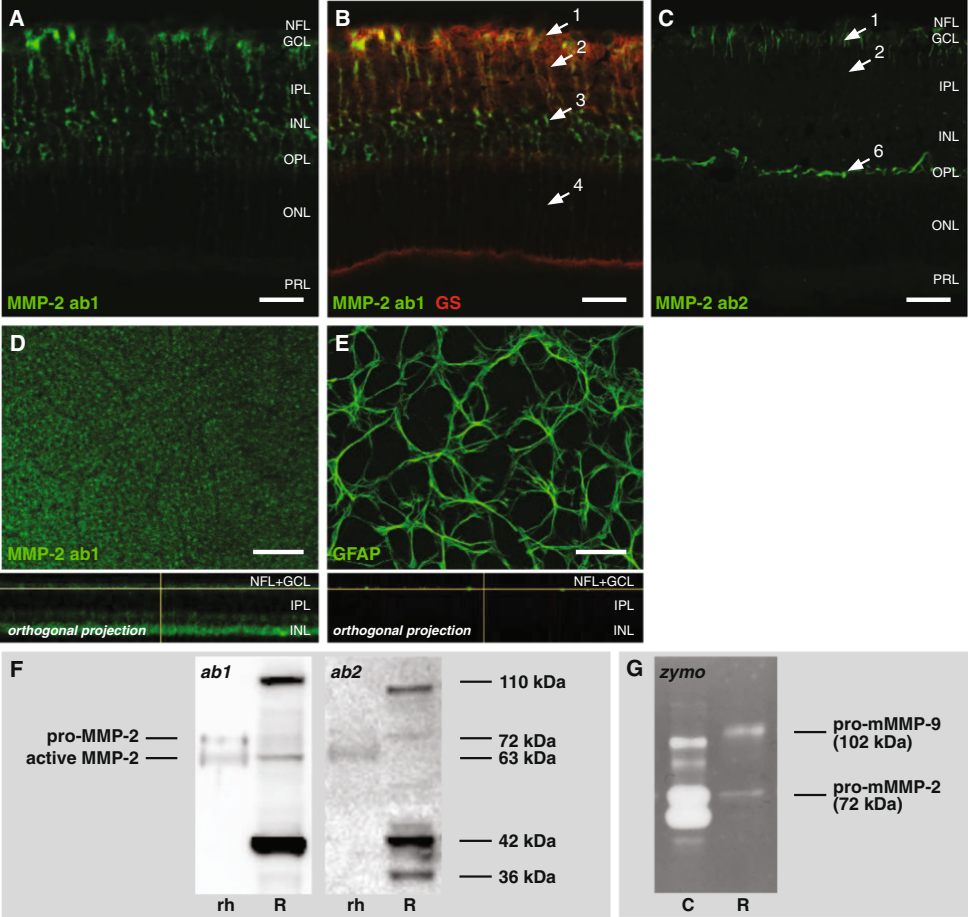
Expression of MMP-2 in the healthy adult mouse retina. **a** Immunostaining with ab1 revealed a macroglial staining pattern for MMP-2 on retinal sections. **b** A double staining for glutamine synthetase (GS) disclosed strong MMP-2 expression in the Müller glia end feet (*arrow 1*), in their somata (*arrow 3*) and radial processes spanning the inner retina (*arrow 2*), as well as a more faint expression in the radial processes spanning the outer retina (*arrow 4*) and in the Müller glia villi (*arrow 5*). **c** Immunostaining with ab2 was predominantly observed in Müller glia end feet (*arrow 1*), in their radial processes in the inner retinal layers (*arrow 2*), and in synaptic sheets in the OPL (*arrow 6*). Scale bars, 20 μm. **d-e** Immunostaining with ab1 on retinal flatmounts revealed that MMP-2 is not expressed by astrocytes, as staining patterns of MMP-2 and GFAP (for astrocytes) were clearly divergent. Rather, MMP-2 expression at the inner retinal surface was seen in a punctuate organization, suggestive of Müller glia end feet. Scale bars, 50 μm. **f** Western blotting on naive retinal tissue lysates (R) with ab1 and ab2 showed that both antibodies recognize the bands corresponding to pro-MMP-2 (72 kDa) and active MMP-2 (63 kDa), as well as an active MMP-2 fragment (42 kDa) and a 110 kDa band. The 36 kDa band visualized by ab2 might represent an autocatalytic cleavage fragment of MMP-2. Recombinant human MMP-2 (rh) was loaded as a positive control. **g** Gelatin zymography on naive mouse retinal tissue lysates (R) revealed pro-MMP-2 (72 kDa) and pro-MMP-9 (102 kDa), yet no active gelatinases. Culture medium from HT1080 human firbosarcoma cells (**c**), containing pro-MMP-9 (92 kDa), active MMP-9 (82 kDa), pro-MMP-2 (72 kDa) and active MMP-2 (63 kDa), was loaded as a positive control.

Immunohistochemical stainings, with both antibodies tested (Fig. 1a–c), revealed that Müller glia express MMP-2 in the adult, healthy mouse retina, as confirmed via double labeling with glutamine synthetase (Fig. 1b). Immunostaining for MMP-2 and GFAP on retinal flatmounts excluded expression of MMP-2 by astrocytes (Fig. 1d-e). With ab1, intense labeling was seen in the end feet of the Müller cells, forming part of the inner limiting membrane, in the radial fibers spanning the entire retina, in the somata in the inner nuclear layer (INL), and -however more faintly- also in the apical villi. Immunostaining with antibody ab2 was less intense, but nevertheless revealed a clear MMP-2 immunopositive signal in the Müller glia end feet and radial fibers in the inner retinal layers. Of note, upon immunostaining with antibody ab2, an intense signal was seen in the outer plexiform layer (OPL) (Fig. 1c). This likely represents labeling of the so-called ‘perisynaptic sheets’ of the Müller cells, responsible for glutamate recycling at the ribbon synapses between photoreceptor and bipolar/horizontal cells [29].

Both antibodies revealed a similar pattern of bands after Western blot on retinal tissue homogenates, although they differentially labeled pro-MMP-2 (72 kDa) and active MMP-2 (63 kDa). While ab1 suggested that MMP-2 is predominantly present in its active form, ab2 indicated a constitutive expression of pro-MMP-2 in the naive retina (Fig. 1f). Gelatin zymography on the same retinal lysates indicated that MMP-2 is merely present in its pro-form, thus corroborating the result obtained with ab2 (Fig. 1g). Additionally, both antibodies labeled a prominent 42 kDa band corresponding to the catalytic domain of MMP-2 [30], as well as a 110 kDa band, which is described to represent MMP-2 homodimers and/or MMP-2-TIMP-2 complexes [30, 31] (Fig. 1f). In contrast to ab1, ab2 recognized an additional band of 36 kDa, which has been previously suggested to be the result of autocatalytic cleavage of the N-terminal propeptide from the 42 kDa MMP-2 fragment [32] (Fig. 1f, right panel).

### MMP-3 expression in the healthy, adult mouse retina

To explore expression and localization of MMP-3, or stromelysin-1, two antibodies were used: a monoclonal rabbit anti-MMP-3 antibody (ab52915, Abcam), further referred to as ab3, and a polyclonal rabbit anti-MMP-3 antibody (sc-6839-R, Santa Cruz), referred to as ab4. For both antibodies, epitopes were mapped at the C-terminal region of the human MMP-3, *i.e.* the hemopexin domain.

**Fig. 2:**
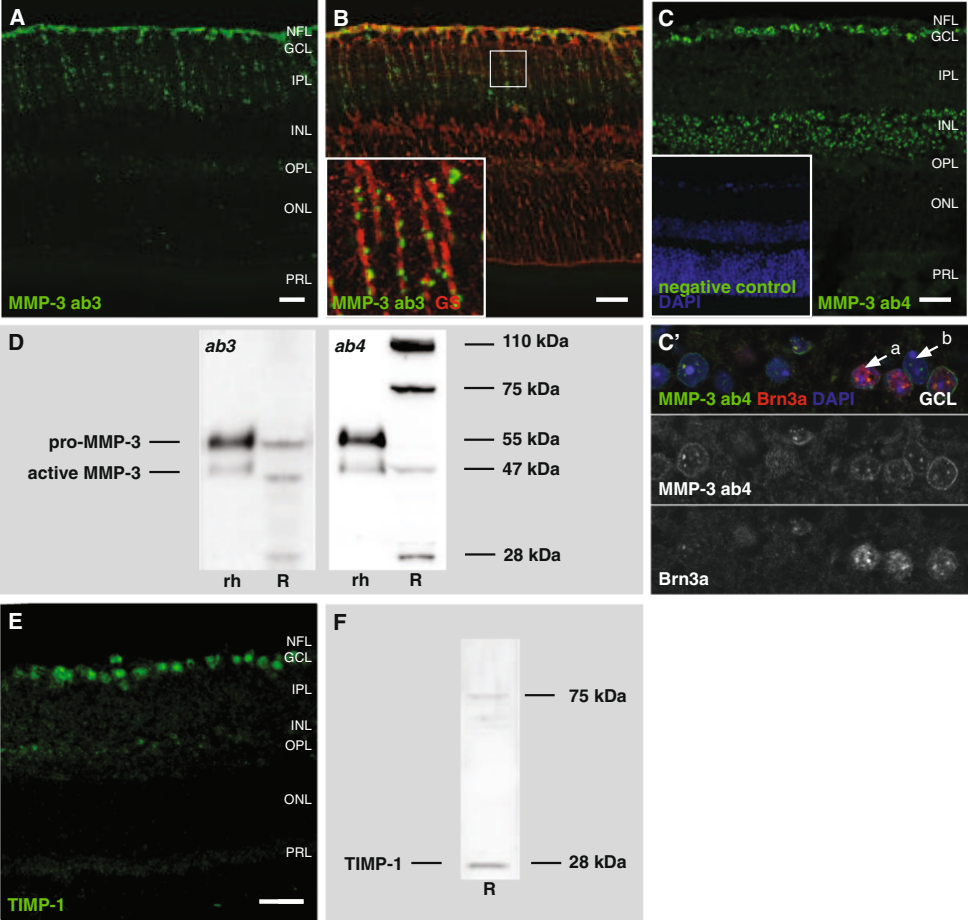
Expression of MMP-3 in the healthy adult mouse retina. **a** Immunostaining with ab3 revealed a punctate expression pattern in all retinal layers, with the most prominent expression in the inner retina. **b** Double labeling for MMP-3 (ab3) and glutamine synthetase (GS) indicated a close association of these MMP-3-positive structures with the radial processes of Müller glia. **c** Immunostaining with ab4 revealed MMP-3 expression in neurons in the GCL and INL. No staining at all was seen on negative control sections (insert). **c’** The magnification images show a double staining with Brn3a, indicating that MMP-3 is expressed by Brn3a^+^ RGCs (*arrow a*) as well as Brn3a^−^ displaced amacrine cells (*arrow b*). Scale bars, 20 μm. **d** Western blotting on naive retina tissue lysates (R) revealed that both antibodies recognized pro-MMP-3 (55 kDa) and active MMP-3 (47 kDa). In addition, ab4 also abundantly labeled free TIMP-1 (28 kDa) and MMP-3-TIMP-1 complexes (75 kDa), and a band around 110 kDa. Recombinant human MMP-3 (rh) was loaded as a positive control. **e** Immunostaining with antibody sc-5538-R showed high TIMP-1 expression in the GCL and more faint expression in the INL and the ECM of both plexiform layers. Scale bar, 20 μm. **f** Western blotting for TIMP-1 on naive retina tissue lysates (R) revealed 28 kDa unbound TIMP-1, as well as a 75 kDa complex of TIMP-1 and MMP-3.

Immunolabeling for MMP-3 with ab3 revealed a diffuse, dotted expression pattern in all layers of the retina (Fig. 2a). Given the size and distribution of these immunopositive puncta, in close association with the radial processes of Müller glia (Fig. 2b), we suggest that MMP-3 might be present in trafficking vesicles. Of note, although the intense labeling in the nerve fiber layer (NFL) likely localized to Müller glia end feet, expression of MMP-3 in astrocytes cannot be excluded. In contrast, immunostaining with the widely used ab4 revealed a distinct MMP-3 expression pattern and showed labeling in (nearly) all neurons in the GCL and interneurons in the INL (Fig. 2c). In the GCL, where immunolabeling was seen in both Brn3a^+^ and Brn3a^−^ neurons, this implies that MMP-3 is expressed by both retinal ganglion cells and displaced amacrine cells (Fig. 2c’, arrow a).

On Western blot, ab3 detected both pro-MMP-3 (55 kDa) and active MMP-3 (47 kDa), while ab4 only labeled the active form of MMP-3 in retinal homogenates (Fig. 2d). In addition, ab4 also abundantly labeled bands with a molecular weight of 28 kDa, 75 kDa and 110 kDa (Fig. 2d, right panel). Reports depicting a 110 kDa band on MMP-3 Western blot and casein zymography [33, 34], along with its theoretical molecular weight of 110 kDa, suggest that this band might represent MMP-3 homodimers. Comparison of the MMP-3 ab4 Western blot with a Western blot for TIMP-1 on identical samples (Fig. 2f), on the other hand, revealed that the 28 kDa band likely represents free TIMP-1 and the 75 kDa band MMP-3-TIMP-1 complexes. Moreover, an immunostaining for TIMP-1 displayed high similarities to the MMP-3 immunostaining with ab4 (Fig. 2e). These findings demonstrate that ab4 is not specific for MMP-3, and indicate a high degree of cross-reactivity against TIMP-1. Importantly, this has strong implications on the interpretation of the immunohistochemical staining for MMP-3 with ab4. Indeed, given the very intense labeling of TIMP-1 (complexes) on Western blot, it can be expected that the observed immunostaining rather represents TIMP-1, or at least both TIMP-1 and MMP-3. Of note, a faint 28 kDa band is also observed on Western blots with ab3, indicating that also here a slight affinity for TIMP-1 is present (Fig. 2d, left panel).

### MMP-9 expression in the healthy, adult mouse retina

In order to study expression and tissue distribution of MMP-9, or gelatinase B, two antibodies from Abcam were tested: a monoclonal mouse anti-MMP-9 antibody (ab58803), referred to as ab5 in the remaining text, and a polyclonal rabbit anti-MMP-9 antibody (ab38898), referred to as ab6. Ab5 recognizes amino acid residues 626–644 of the human MMP-9, which are part of the hemopexin domain, while ab6 was produced via immunization with full-length mouse MMP-9.

**Fig. 3:**
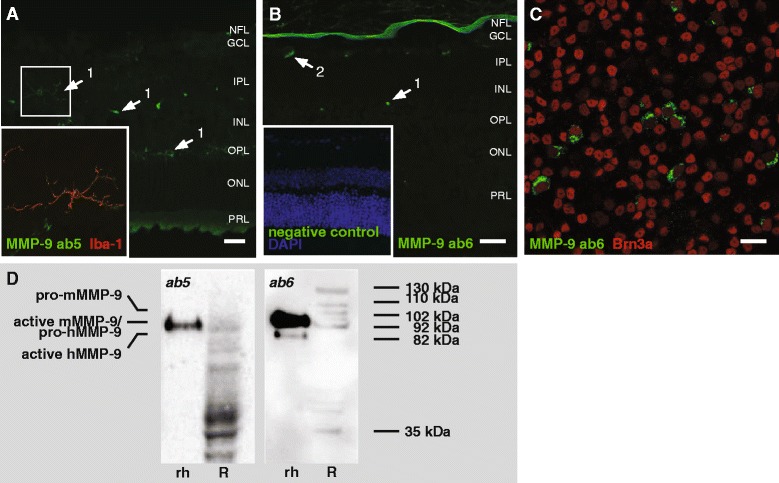
Expression of MMP-9 in the healthy, adult mouse retina. **a** Immunostaining with ab5 revealed MMP-9 in resident microglia (*arrow 1*), predominantly in the IPL, INL and OPL. The insert clearly shows co-localization of MMP-9 and Iba-1. **b** Immunostaining with ab6 labeled MMP-9 expression in the small cell bodies of microglia in the INL (*arrow 1*), but MMP-9 was also found to be expressed in a handful of RGCs (*arrow 2*). Of note, the strong immunoreactivity of the inner limiting membrane might be related to the antibody’s affinity for fibronectin-like domains, which are not only present in MMP-9, but also in the basal lamina of the inner limiting membrane. No staining at all was seen on negative control sections (insert). **c** A double staining for MMP-9 (*ab6*) and Brn3a on a retinal flatmount disclosed MMP-9 expression by a subset of RGCs. Scale bars, 20 μm. **d** Western blotting on naive retina tissue lysates (*R*) with ab5 disclosed active MMP-9 (92 kDa) and an active MMP-9 fragment (35 kDa). In addition, a series of unknown bands was seen. Western blotting with ab6, revealed both pro-MMP-9 (102 kDa) and active MMP-9 (92 kDa), as well as MMP-9 complexes (110 and 130 kDa) and an active MMP-9 fragment (35 kDa). Recombinant human MMP-9 (rh) was loaded as a control. Of note, while mouse pro- and active MMP-9 have a molecular weight of 102 kDa and 92 kDa, human pro- and active MMP-9 weigh 92 kDa and 82 kDa, respectively.

As evidenced by a double staining with Iba-1, ab5 stained quiescent microglia -sometimes with elaborate ramifications- present in the inner plexiform layer (IPL), INL and OPL (Fig. 3a). Correspondingly, ab6 revealed MMP-9 expression in microglia in the INL, but also labeled scarce neurons in the ganglion cell layer (GCL) (Fig. 3b–c). Subsequent double stainings for MMP-9 and Brn3a on retinal flatmounts further disclosed that MMP-9 is indeed expressed by a minority of RGCs in the adult healthy retina (Fig. 3c), as already described in literature [35, 36]. Of note, ab5 also labeled RGCs, but only upon injury when they upregulate their MMP-9 expression (data not shown). In addition, ab6 also intensely labeled the inner limiting membrane, however, it remains speculative whether this represents specific immunolabeling of MMP-9. Indeed, the presence of fibronectin-like domains within the catalytic domain of MMP-9, might be underlying cross-reactivity of ab6 for this basal lamina constituent (Fig. 3b).

Western blotting on mouse retinal tissue lysates revealed that the healthy, murine retina contains very low amounts of MMP-9, adding to the difficulties to accurately discern MMP-9 on Western blot. Nevertheless, both antibodies appeared to recognize active mouse MMP-9 (92 kDa), as well as a 35 kDa band, likely corresponding to an active MMP-9 subspecies resulting from autocatalytic processing [37] (Fig. 3d). In addition, and in contrast to ab5, ab6 also detected pro-MMP-9 (102 kDa) and previously described MMP-9-TIMP-1 and MMP-9- neutrophil gelatinase B-associated lipocalin complexes (110–130 kDa) [38–40] (Fig. 3d, right panel). Ab5 on the other hand labeled a smear of bands with descending molecular weights from 82 kDa to 30 kDa, of which the identity is unknown (Fig. 3d, left panel). Notably, while mouse MMP-9 exists as a 102 kDa pro-form and a 92 kDa active form, human pro- and active MMP-9 weight 92 kDa and 82 kDa, respectively.

### MT1-MMP expression in the healthy, adult mouse retina

To study the expression pattern of MT1-MMP, or MMP-14, two polyclonal rabbit anti-MT1-MMP antibodies were evaluated. According to the manufacturers, antibody ab53712 (Abcam) (referred to as ab7) recognizes the hemopexin domain, more specifically amino acid residues 471–520 of human MT1-MMP, while antibody RP-3 (Triple Point) (referred to as ab8) specifically recognizes the catalytic domain of MT1-MMP.

**Fig. 4:**
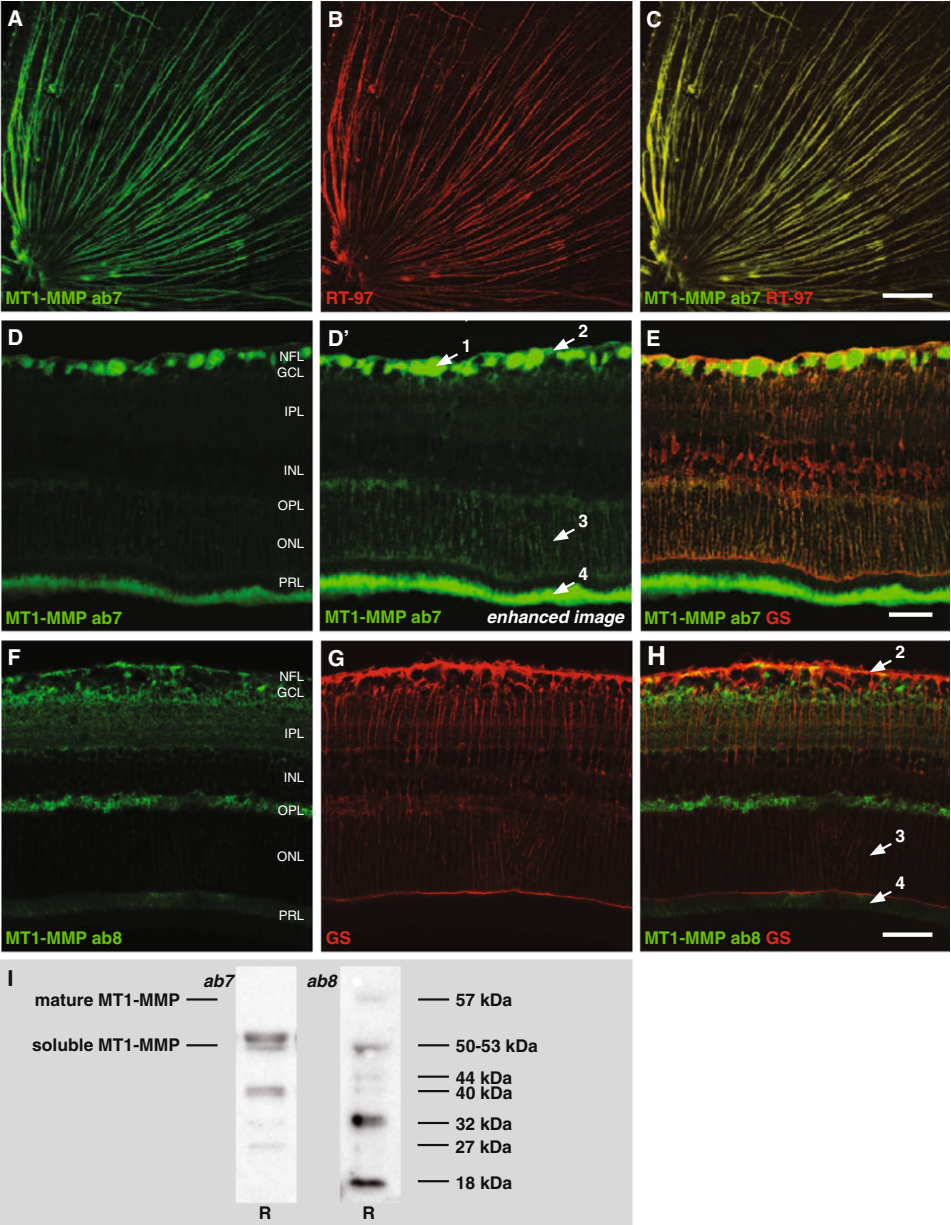
Expression of MT1-MMP in the healthy adult mouse retina. **a** Immunostaining with ab7 on a flatmounted retina revealed that MT1-MMP is expressed by all RGC axons. **b-c** The axonal labeling was confirmed by a double staining for phosphorylated neurofilament heavy (detected with the RT-97 antibody). Scale bar, 200 μm. **d-d’** In addition to this abundant expression by RGC axons, MT1-MMP expression is also seen in the Müller glia with ab7. Of note, glial expression levels of MT1-MMP are lower than in the axons, and image exposure time was lengthened to reveal this more faint MT1-MMP expression. **e** A double staining with glutamine synthetase (GS), confirmed that – besides expression in the axons (*arrow 1*) –, ab7 also visualized MT1-MMP expression in Müller glia end feed (*arrow 2*) and radial processes (*arrow 3*) and in photoreceptor outer segments (*arrow 4*) on retinal sections. Scale bar, 20 μm. **f** Immunostaining with ab8 confirmed MT1-MMP expression in the Müller glia end feet (*arrow 2*) and radial processes (*arrow 3*) and in photoreceptor inner segments (*arrow 4*), but did not reveal axonal expression. **g-h** MT1-MMP expression in Müller glia was confirmed by a double staining with glutamine synthetase. Scale bar, 20 μm. **i** Western blotting with antibody ab7 revealed soluble, active MT1-MMP (50–53 kDa), in addition to several MT1-MMP subspecies (40 kDa, 32 kDa, 27 kDa). Western blotting with ab8 revealed membrane-bound, active MT1-MMP (57 kDa), as well as soluble, active MT1-MMP (50 kDa) and 44 kDa, 40 kDa, 32 kDa and 18 kDa fragments.

Immunostaining with antibody ab7 in the retina and optic nerve head of healthy adult mice, disclosed high MT1-MMP expression in the RGC axon bundles (Fig. 4a–c), and immunostaining on brain sections also revealed axonal MT1-MMP expression in the optic nerve and primary retinal target areas in the brain, *i.e.* the lateral geniculate nucleus and the superior colliculus (data not shown). In addition, although at lower levels as compared to the axonal expression, MT1-MMP immunostaining was observed in Müller glia, with the highest intensity seen in the end feet in the GCL and NFL and in the radial fibers spanning the outer retinal layers (Fig. 4d–f). Also ab8 labeled end feet and radial fibers of the Müller cells, yet it did not label RGC axons (Fig. 4g–h). Of note, MT1-MMP expression was also observed in the photoreceptors, however, while ab7 stained the outer segments, ab8 labeled the inner segments.

Western blotting showed that, while both antibodies recognized soluble, active MT1-MMP (50–53 kDa), only ab8 labeled membrane-bound, active MT1-MMP (57 kDa) (Fig. 4i). In addition, both antibodies revealed a series of bands that represent MT1-MMP subspecies resulting from autocatalytic and non-autocatalytic processing and shedding of 57 kDa MT1-MMP [41]. Ab7 labeled protein bands of 40 kDa (40–44 kDa membrane-anchored, inactive degradation product), 32 kDa and 27 kDa (27–32 kDa active soluble fragment) (Fig. 4i, right panel), while ab8 recognized protein bands of 44 kDa, 40 kDa, 32 kDa and 18 kDa (inactive, soluble fragment) (Fig. 4i, left panel). Of note, it is theoretically impossible for ab8, which has been designed to recognize the catalytic domain, to recognize the 40–44 kDa inactive subspecies of MT1-MMP, as these lack the catalytic domain. This raises concerns about the specificity of ab8.

## Discussion

In this manuscript, the baseline spatial expression pattern of a selection of MMPs, namely MMP-2, -3, -9 and MT1-MMP, was investigated in the adult mouse retina. Immunohistochemistry with commercially available MMP antibodies was combined with double stainings with validated antibodies against retinal cell markers, to identify the cell types that express these MMPs. Experience has learned that the specificity of antibodies, commercially available or self-made, is not guaranteed. Therefore, the antibodies were validated by performing Western blot on retinal tissue lysates and recombinant MMPs to check for their specificity and sensitivity.

### MMP-2 expression in the healthy, adult mouse retina

Both antibodies under study revealed MMP-2 expression in the Müller glia, although there were differences in the amount and localization of the staining: while the use of ab1 resulted in immunostaining of the entire Müller cell, ab2 only stained the Müller cell compartments in the inner retinal layers. Likely, MMP-2 expression is more abundant in the inner retinal layers and the observed difference reflects a difference in sensitivity between both antibodies. Moreover, gelatin zymography data indicated that ab2 is likely to more reliably discriminate between pro-MMP-2 *versus* active MMP-2 than ab1, as both gelatin zymography and Western blot with ab2 agree that MMP-2 is present in its pro-form in the naive retina. Moreover, ab1 performed poorly on PFA-fixed cryosections so that unfixed cryosections are needed for good results, which is a major drawback in terms of histological and morphological quality. As a consequence, we propose ab2 for further studies. Of note, while there was only faint staining of pro- and active MMP-2, high molecular weight complexes and low molecular weight degradation products were abundantly labeled on the MMP-2 Western blots, indicating that there is a high turnover of MMP-2 in the healthy adult mouse retina.

When comparing these results to what is known about MMP-2 expression in literature, it turns out that our present immunohistochemical data largely confirm previous MMP-2 expression data. The sole publication describing MMP-2 expression in the mouse retina revealed MMP-2 immunoreactivity in Müller glia and astrocytes, in addition to RGCs [42]. In primates, more specifically in human and macaque monkey, MMP-2 expression was also detected in RGC somata and in their axons in the NFL, however no glial expression was observed [43, 44]. Given this very limited amount of data to compare with, it remains speculative whether MMP-2 expression is to be expected in RGCs and/or their axons. To answer this question, the antibody used by Zhang et al. [42] should be evaluated for specificity, similar to the validation of the antibodies performed here, and double stainings should be performed to irrefutably demonstrate expression by RGCs and astrocytes. Alternative strategies to study spatial expression patterns of MMP-2, include *in situ* hybridization and *in situ* gelatin zymography. However, while the first technique is limited to the visualization of mRNA and thus does not reveal protein expression nor activity, the second is not specific for MMP-2 as it visualizes all gelatinolytic activity.

Current knowledge about the role of MMP-2 in the healthy *versus* glaucomatous retina is as sparse as our knowledge about its expression. MMP-2 activity/expression was reported to remain unchanged in some reports [35, 42, 43, 45, 46], while others -although using the same GON models- revealed increased MMP-2 activity within the first hours post injury [42, 47]. Based on its spatial expression pattern described here, one can hypothesize that MMP-2 might be involved in glial reactivity upon retinal damage. Nevertheless, MMP-2 deficiency did not protect from RGC death after optic nerve ligation in mice [23].

### MMP-3 expression in the healthy, adult mouse retina

The results obtained via immunohistochemistry with the two MMP-3 antibodies under study, appeared very contradicting at first sight. Indeed, while ab3 revealed a macroglial staining pattern, ab4 clearly indicated that MMP-3 was expressed by neurons. However, an explanation for these puzzling data was found in the Western blot patterns of the antibodies. Ab3 detected both pro-MMP-3 and active MMP-3. Ab4, on the contrary, weakly labeled active mouse MMP-3 but no pro-MMP-3, yet abundantly labeled protein bands of 28 kDa, 75 kDa and 110 kDa. Remarkably, the protein bands of 28 kDa and 75 kDa were identified as free TIMP-1 and a MMP-3-TIMP-1 complexes, respectively. Of note, also ab3 revealed a faint band of 28 kDa, indicating that also this antibody is not entirely specific for MMP-3 and might recognize free TIMP-1 as well. In line with these results, Western blot and immunohistochemistry experiments with these two antibodies in the developing cerebellum, unveiled a similar discrepant neuronal *versus* glial expression pattern, as well as additional Western blot bands [12].

The limited specificity of the MMP-3 antibodies has major implications for their use in immunohistochemical stainings. The use of ab4 resulted in immunolabeling of displaced amacrine cells and RGCs in the GCL, as well as interneurons in the INL. However, it can be assumed that TIMP-1 expression, rather than (or in addition to) MMP-3 expression, was visualized. Ab3 revealed punctate MMP-3-immunopositive structures along the radial fibers of the Müller glia, and, notwithstanding its weak affinity for free TIMP-1, seems to be a reliable antibody to detect MMP-3 protein expression in the retina. The nature of these presumed vesicles, which are present throughout all retinal layers but are most abundant in the inner retina, remains thus far unidentified.

Limited attention has been devoted to the expression and function of MMP-3 in the healthy and glaucomatous retina so far. The basal expression pattern of MMP-3 has not been described yet, although a strong increase in MMP-3 mRNA expression in the retina of rats subjected to an ocular hypertension GON model or to optic nerve injury [48–50] suggests MMP-3 to be an important player during glaucomatous damage. The glial origin of MMP-3 expression discovered here could indicate the involvement of MMP-3 in glial reactivity. On the other hand, the MMP-3 loaded vesicles within the Muller glia can be transported along the entire retina and therefore be involved in virtually every pathophysiological process, not to mention the described pro- and anti-apoptotic actions of MMP-3 in the CNS [12, 51, 52].

### MMP-9 expression in the healthy, adult mouse retina

Despite the striking differences in their Western blots, a highly similar immunostaining pattern was seen with the two anti-MMP-9 antibodies under study. Both antibodies indicated that quiescent microglia are the most important MMP-9-expressing cells in the healthy, adult mouse retina. In addition, a very limited number of RGCs was visualized upon immunohistochemical staining with ab6. Of note, while no MMP-9 expression in RGCs was seen with antibody ab5 in the naive retina, abundant RGC staining could be observed in retinas of mice subjected to a GON model [53]. This is in line with existing literature, which states that MMP-9 expression is upregulated in RGCs undergoing apoptosis [23, 24, 35, 36, 42, 45–47, 54]. As the number of dying RGCs is almost non-existing in a naive retina and the expression level of MMP-9 very low, this might be the reason why there was no RGC immunostaining seen with antibody ab5, hence indicating that ab5 is likely to have a lower sensitivity as compared to antibody ab6. Moreover, ab5 only recognized active mouse MMP-9 on a Western blot, while ab6, designed to recognize full-length mouse MMP-9, revealed both pro-MMP-9 and active MMP-9.

Remarkably, the use of ab6 for MMP-9 immunostaining also resulted in an intense labeling of the inner limiting membrane. As ab6 was generated by immunization with full length MMP-9, which includes three contiguous fibronectin type II-like domains within the catalytic domain, there is a high chance that this is the result of cross-reactivity of ab6 with fibronectin, one of the major constituents of the inner limiting membrane. Nevertheless, Western blot with ab6 on retinal tissue homogenates indicated that this is a highly specific antibody for MMP-9, as all protein bands were related to MMP-9 subspecies, complexes and degradation products. The Western blot with ab5, on the other hand, only revealed active MMP-9 in the mouse retina, in addition to a series of protein bands with unknown identity. Notably, Western blot labeling patterns of MMP pro- and active subspecies seem not readily translatable between mice and human samples. As illustrated by this Western blot for MMP-9, antibodies recognizing pro- and/or active MMP in mouse retinal samples do not necessarily also recognize pro- and/or active recombinant human MMP. Taken together, based on these Western blot data, ab6 seems to be the most reliable antibody, even despite its potential affinity for fibronectin.

A multitude of studies, using various rodent models of GON, have revealed that MMP-9 expression and activity in the GCL increases in the glaucomatous retina [23, 24, 35, 36, 42, 45–47, 54] and that this increased MMP-9 activity plays a key role in the promotion of detachment-induced RGC death by interfering with integrin-mediated survival signaling [24, 36, 55]. Using one particular antibody for immunohistochemistry, two studies pointed out reactive astrocytes as the source of MMP-9 expression, both in the naive and glaucomatous mouse retina [42, 54], and excluded endothelial cells, microglia and RGCs [54]. Other studies used *in situ* gelatin zymography and immunohistochemistry to point out that MMP-9 expression is virtually absent in the naive retina but upregulated in RGCs upon induction of glaucomatous damage [35, 36]. The findings obtained within this chapter seem to support the latter theory, although we also revealed MMP-9 expression by microglia.

### MT1-MMP expression in the healthy, adult mouse retina

MT1-MMP is a very complex MMP to investigate. Not only does this MMP occur in its membrane-bound form, it also undergoes autocatalytic and non-autocatalytic processing events, resulting in a series of MT1-MMP subspecies with varying degrees of activity, which can be endocytosed from and/or recycled back to the cell surface and shed into the extracellular space [41, 56]. Both antibodies tested appear to recognize many of these subspecies, which resulted in very complex Western blot patterns. Western blotting of retinal tissue homogenates pointed out that both antibodies labeled the active, soluble form of MT1-MMP (50–53 kDa) that is shed from the cell membrane upon cleavage in the stem region of mature MT1-MMP, while the active, membrane-bound MT1-MMP (mature MT1-MMP, 57 kDa) was only recognized by ab8. In addition, both antibodies revealed MT1-MMP processing/shedding products. It is, however, most clear that they have very different affinities: while ab7 preferentially labeled soluble MT1-MMP (50–53 kDa), ab8 most abundantly labeled 27–32 kDa fragments. Notably, these 27–32 kDa subspecies have been identified as a soluble, catalytically competent products formed by non-autocatalytic shedding of 57 kDa membrane-bound MT1-MMP. Due to the complexity and incomplete overlap of the labeling patterns of both antibodies, it remains unclear which one is preferable over the other. While we argue that ab8 may show a lack of specificity (see above), ab7 does not label membrane-bound mature MT1-MMP and hence overlooks the physiologically most relevant MT1-MMP species. The best choice might be dependent on the scientific question to be answered in a certain Western blot or immunohistochemistry experiment.

When applied for immunohistochemistry, both antibodies unequivocally revealed MT1-MMP expression by Müller glia. MT1-MMP immunostaining was very clear in the NFL and GCL, where the end feet of the Müller glia were highly immunoreactive, moreover, ab7 also visualized MT1-MMP-positive Müller glia radial fibers in the outer retinal layers. Second, MT1-MMP was also found in the light-sensitive segments of the photoreceptors, namely in the outer segments when stained with ab7 and in the inner segments when immunolabeled using ab8. Finally, ab7 intensely labeled RGC axon bundles in the NFL. As it can be assumed, based on the validation by Western blot, that both antibodies are specific for MT1-MMP, it is striking that ab7 did label RGC axons while ab8 did not. One could speculate that MT1-MMP subspecies are present in different cell types/cell compartments, resulting in differential labeling by these antibodies due to their differential specificity/sensitivity for certain subspecies of MT1-MMP.

Expression data about MT1-MMP in the retina is very limited, especially in mice. One report, in which MT1-MMP expression was studied in the retina of newborn mice (P0) by means of *in situ* hybridization, described MT1-MMP expression in the NFL [57]. Higher resolution images and immunohistochemical double stainings are necessary to determine whether MT1-MMP mRNA was seen in axons and/or in Müller glia end feet there. Moreover, *in situ* hybridization images shown in this manuscript also revealed MT1-MMP mRNA expression in the photoreceptor layer (PRL). Likewise, MT1-MMP expression was seen in the photoreceptor inner and outer segments of human and equine retina, respectively [58, 59]. Immunohistochemical staining for MT1-MMP on rabbit retinal sections resulted in labeling of the inner retinal layers [60], displaying high similarity to the staining pattern of Müller glia end feet. On the contrary, immunohistochemical stainings and *in situ* hybridization experiments in human and monkey optic nerves, did not reveal MT1-MMP expression in axons, but even so in astrocytes [43, 44]. Taken together, the data presented in this chapter are in line with the limited expression data available in mouse and rabbit, but do not correspond to the expression profile that was observed in primates.

Remarkably, virtually nothing is known about the role of MT1-MMP in the retina. Based on its spatial expression pattern described here, one could speculate that MT1-MMP might be involved in maintaining axonal integrity and/or in glial reactivity. However there is no concrete evidence to support these theories and further studies exploring its function in the retina are urgently needed.

## Conclusions

In summary, the basal expression pattern of MMP-2, -3, -9 and MT1-MMP was studied in the mouse retina by means of immunohistochemical stainings and Western blot. All four MMPs were found to be expressed in the retina of healthy, adult mice. MMP-2 expression was seen in Müller glia, predominantly in their end feet, which is in line with available literature. MMP-3 expression was described for the first time in the retina, and was observed in vesicle-like structures along the radial fibers of Müller glia. MMP-9 expression has already extensively been studied but still discords exists about its cellular source of expression. The data obtained in this study corroborate that MMP-9 is expressed by microglia and by a sparse subset of (apoptosing) RGCs. MT1-MMP localization was for the first time studied in adult mice and was found in RGC axons and Müller glia, mimicking the MT1-MMP expression pattern seen in rabbits and neonatal mice.

For each of the MMP antibodies, Western blots were performed to evaluate their specificity and sensitivity. Results indicated that the specificity of an antibody cannot be taken for granted, and that immunostainings should always be interpreted with caution. Nevertheless, one antibody was selected for each MMP, based on its staining pattern in WB. Notably, these Western blot experiments further emphasized the complex regulation of MMPs: pro-form *versus* active MMPs were seen, as well as degradation products and homo- and heterodimers. This adds an extra level of complexity to the interpretation of immunohistochemical stainings and Western blots, and, once again, stresses the importance of using complementary sets of well-validated techniques to evaluate MMP expression and activity.

Altogether, these data can be instrumental to study MMP expression in mouse models of retinal pathologies. Indeed, current literature about MMPs in the retina, and their potential contribution to retinal pathologies, is very limited. Nevertheless, the combined data from expression studies in glaucoma patients and animal models of GON point to the involvement of MMPs during retinal neurodegeneration. A first requisite for the disentanglement of the exact role of MMPs in GONs, and other retinal/ocular pathologies, is to assess whether MMP expression in animal disease models corresponds to what has been observed in clinical studies.

## Abbreviations

CNS: Central nervous system
ECM: Extracellular matrix
FITC: Fluorescein isothiocyanate
GCL: Ganglion cell layer
GON: Glaucomatous optic neuropathy
HRP: Horseradish peroxidase
INL: Inner nuclear layer
IPL: Inner plexiform layer
MMP: Matrix metalloproteinase
NFL: Nerve fiber layer
OPL: Outer plexiform layer
P0: Postnatal day 0
PBS: Phosphate-buffered saline
PFA: Paraformaldehyde
PRL: Photoreceptor layer
RGC: Retinal ganglion cell
TBS: Tris-buffered saline
TIMP: Tissue inhibitor of matrix metalloproteinase

## References

[1] Butler GS, Overall CM (2009). Updated biological roles for matrix metalloproteinases and new “intracellular” substrates revealed by degradomics. Biochemistry.

[2] Morrison CJ, Butler GS, RodrÃ­guez D, Overall CM (2009). Matrix metalloproteinase proteomics: substrates, targets, and therapy. Curr Opin Cell Biol.

[3] Rodríguez D, Morrison CJ, Overall CM (2010). Matrix metalloproteinases: What do they not do? New substrates and biological roles identified by murine models and proteomics. Biochimica et Biophysica Acta (BBA) - Molecular Cell Research.

[4] Agrawal SM, Lau L, Yong VW (2008). MMPs in the central nervous system: where the good guys go bad. Semin Cell Dev Biol.

[5] De Groef L, Van Hove I, Dekeyster E, Stalmans I, Moons L (2014). MMPs in the neuroretina and optic nerve: modulators of glaucoma pathogenesis and repair?. Invest Ophthalmol Vis Sci.

[6] Duchossoy Y, Arnaud S, Feldblum S (2001). Matrix metalloproteinases: potential therapeutic target in spinal cord injury. Clin Chem Lab Med.

[7] Johri A, Beal MF (2010). Hunting-ton for new proteases: MMPs as the new target?. Neuron.

[8] Lee S-R, Tsuji K, Lee S-R, Lo EH (2004). Role of Matrix Metalloproteinases in Delayed Neuronal Damage after Transient Global Cerebral Ischemia. J Neurosci.

[9] Rosenberg GA (2009). Matrix metalloproteinases and their multiple roles in neurodegenerative diseases. Lancet Neurol.

[10] Yong VW, Zabad RK, Agrawal S, Goncalves Dasilva A, Metz LM (2007). Elevation of matrix metalloproteinases (MMPs) in multiple sclerosis and impact of immunomodulators. J Neurol Sci.

[11] Zhang H, Adwanikar H, Werb Z, Noble-Haeusslein LJ (2010). Matrix metalloproteinases and neurotrauma: evolving roles in injury and reparative processes. Neuroscientist.

[12] Van Hove I, Lemmens K, Van de Velde S, Verslegers M, Moons L (2012). Matrix metalloproteinase-3 in the central nervous system: a look on the bright side. J Neurochem.

[13] Verslegers M, Lemmens K, Van Hove I, Moons L (2013). Matrix metalloproteinase-2 and -9 as promising benefactors in development, plasticity and repair of the nervous system. Prog Neurobiol.

[14] Dzwonek J, Rylski M, Kaczmarek L (2004). Matrix metalloproteinases and their endogenous inhibitors in neuronal physiology of the adult brain. FEBS Lett.

[15] Milward EA, Fitzsimmons C, Szklarczyk A, Conant K (2007). The matrix metalloproteinases and CNS plasticity: an overview. J Neuroimmunol.

[16] Sivak JM, Fini ME (2002). MMPs in the eye: emerging roles for matrix metalloproteinases in ocular physiology. Prog Retin Eye Res.

[17] Wride MA, Geatrell J, Guggenheim JA (2006). Proteases in eye development and disease. Birth Defects Res C Embryo Today.

[18] Bhatt LK, Addepalli V (2010). Attenuation of diabetic retinopathy by enhanced inhibition of MMP-2 and MMP-9 using aspirin and minocycline in streptozotocin-diabetic rats. Am J Transl Res.

[19] Giebel SJ, Menicucci G, McGuire PG, Das A (2005). Matrix metalloproteinases in early diabetic retinopathy and their role in alteration of the blood-retinal barrier. Lab Invest.

[20] Kowluru RA, Kanwar M (2009). Oxidative stress and the development of diabetic retinopathy: contributory role of matrix metalloproteinase-2. Free Radic Biol Med.

[21] Kowluru RA, Zhong Q, Santos JM (2012). Matrix metalloproteinases in diabetic retinopathy: potential role of MMP-9. Expert Opin Investig Drugs.

[22] Chintala SK (2006). The emerging role of proteases in retinal ganglion cell death. Exp Eye Res.

[23] Chintala SK, Zhang X, Austin JS, Fini ME (2002). Deficiency in matrix metalloproteinase gelatinase B (MMP-9) protects against retinal ganglion cell death after optic nerve ligation. J Biol Chem.

[24] Guo L, Moss SE, Alexander RA, Ali RR, Fitzke FW, Cordeiro MF (2005). Retinal ganglion cell apoptosis in glaucoma is related to intraocular pressure and IOP-induced effects on extracellular matrix. Invest Ophthalmol Vis Sci.

[25] Markiewicz L, Majsterek I, Przybylowska K, Dziki L, Waszczyk M, Gacek M (2013). Gene polymorphisms of the MMP1, MMP9, MMP12, IL-1beta and TIMP1 and the risk of primary open-angle glaucoma. Acta Ophthalmol.

[26] Kaminska A, Banas-Lezanska P, Przybylowska K, Gacek M, Majsterek I, Szaflik J (2014). The protective role of the -735C/T and the -1306C/T polymorphisms of the MMP-2 gene in the development of primary open-angle glaucoma. Ophthalmic Genet.

[27] Micheal S, Yousaf S, Khan MI, Akhtar F, Islam F, Khan WA (2013). Polymorphisms in matrix metalloproteinases MMP1 and MMP9 are associated with primary open-angle and angle closure glaucoma in a Pakistani population. Mol Vis.

[28] Schlotzer-Schrehardt U, Lommatzsch J, Kuchle M, Konstas AG, Naumann GO (2003). Matrix metalloproteinases and their inhibitors in aqueous humor of patients with pseudoexfoliation syndrome/glaucoma and primary open-angle glaucoma. Invest Ophthalmol Vis Sci.

[29] Reichenbach A, Bringmann A (2010). Müller Cells in the Healthy and Diseased Retina.

[30] Newsome AL, Johnson JP, Seipelt RL, Thompson MW (2007). Apolactoferrin inhibits the catalytic domain of matrix metalloproteinase-2 by zinc chelation. Biochem Cell Biol.

[31] Zucker S, Lysik RM, Gurfinkel M, Zarrabi MH, Stetler-Stevenson W, Liotta LA (1992). Immunoassay of type IV collagenase/gelatinase (MMP-2) in human plasma. J Immunol Methods.

[32] Crabbe T, Ioannou C, Docherty AJ (1993). Human progelatinase A can be activated by autolysis at a rate that is concentration-dependent and enhanced by heparin bound to the C-terminal domain. Eur J Biochem.

[33] Métayer S, Dacheux F, Dacheux J-L, Gatti J-L (2002). Comparison, Characterization, and Identification of Proteases and Protease Inhibitors in Epididymal Fluids of Domestic Mammals. Matrix Metalloproteinases Are Major Fluid Gelatinases. Biol Reprod.

[34] Göõz M, Göõz P, Smolka AJ (2001). Epithelial and bacterial metalloproteinases and their inhibitors in H. pylori infection of human gastric cells. American Journal of Physiology - Gastrointestinal and Liver Physiology.

[35] Manabe S, Gu Z, Lipton SA (2005). Activation of matrix metalloproteinase-9 via neuronal nitric oxide synthase contributes to NMDA-induced retinal ganglion cell death. Invest Ophthalmol Vis Sci.

[36] Santos ARC, Corredor RG, Obeso BA, Trakhtenberg EF, Wang Y, Ponmattam J (2012). Beta1 Integrin-Focal Adhesion Kinase (FAK) Signaling Modulates Retinal Ganglion Cell (RGC) Survival. PLoS One.

[37] Ries C, Pitsch T, Mentele R, Zahler S, Egea V, Nagase H (2007). Identification of a novel 82 kDa proMMP-9 species associated with the surface of leukaemic cells: (auto-)catalytic activation and resistance to inhibition by TIMP-1. Biochem J.

[38] Berthier CC, Lods N, Joosten SA, van Kooten C, Leppert D, Lindberg RLP (2006). Differential regulation of metzincins in experimental chronic renal allograft rejection: Potential markers and novel therapeutic targets. Kidney Int.

[39] Kim Y, Remacle AG, Chernov AV, Liu H, Shubayev I, Lai C (2012). The MMP-9/TIMP-1 Axis Controls the Status of Differentiation and Function of Myelin-Forming Schwann Cells in Nerve Regeneration. PLoS One.

[40] Ardi VC, Kupriyanova TA, Deryugina EI, Quigley JP (2007). Human neutrophils uniquely release TIMP-free MMP-9 to provide a potent catalytic stimulator of angiogenesis. Proc Natl Acad Sci U S A.

[41] Toth M, Osenkowski P, Hesek D, Brown S, Meroueh S, Sakr W (2005). Cleavage at the stem region releases an active ectodomain of the membrane type 1 matrix metalloproteinase. Biochem J.

[42] Zhang X, Cheng M, Chintala SK (2004). Kainic acid-mediated upregulation of matrix metalloproteinase-9 promotes retinal degeneration. Invest Ophthalmol Vis Sci.

[43] Agapova OA, Kaufman PL, Lucarelli MJ, Gabelt BT, Hernandez MR (2003). Differential expression of matrix metalloproteinases in monkey eyes with experimental glaucoma or optic nerve transection. Brain Res.

[44] Agapova OA, Ricard CS, Salvador-Silva M, Hernandez MR (2001). Expression of matrix metalloproteinases and tissue inhibitors of metalloproteinases in human optic nerve head astrocytes. Glia.

[45] Sun MH, Chen KJ, Tsao YP, Kao LY, Han WH, Lin KK (2011). Down-regulation of matrix metalloproteinase-9 by pyrrolidine dithiocarbamate prevented retinal ganglion cell death after transection of optic nerve in rats. Curr Eye Res.

[46] Zhang X, Chintala SK (2004). Influence of interleukin-1 beta induction and mitogen-activated protein kinase phosphorylation on optic nerve ligation-induced matrix metalloproteinase-9 activation in the retina. Exp Eye Res.

[47] Zhang X, Sakamoto T, Hata Y, Kubota T, Hisatomi T, Murata T (2002). Expression of matrix metalloproteinases and their inhibitors in experimental retinal ischemia-reperfusion injury in rats. Exp Eye Res.

[48] Ahmed F, Brown KM, Stephan DA, Morrison JC, Johnson EC, Tomarev SI (2004). Microarray analysis of changes in mRNA levels in the rat retina after experimental elevation of intraocular pressure. Invest Ophthalmol Vis Sci.

[49] Agudo M, Perez-Marin MC, Lonngren U, Sobrado P, Conesa A, Canovas I (2008). Time course profiling of the retinal transcriptome after optic nerve transection and optic nerve crush. Mol Vis.

[50] Yang Z, Quigley HA, Pease ME, Yang Y, Qian J, Valenta D (2007). Changes in gene expression in experimental glaucoma and optic nerve transection: the equilibrium between protective and detrimental mechanisms. Invest Ophthalmol Vis Sci.

[51] Kim EM, Hwang O (2011). Role of matrix metalloproteinase-3 in neurodegeneration. J Neurochem.

[52] Kim E-M, Shin E-J, Choi JH, Son HJ, Park I-S, Joh TH (2010). Matrix Metalloproteinase-3 Is Increased and Participates in Neuronal Apoptotic Signaling Downstream of Caspase-12 during Endoplasmic Reticulum Stress. Journal of Biological Chemistry.

[53] De Groef L, Salinas-Navarro M, Van Imschoot G, Libert C, Vandenbroucke RE, Moons L (2015). Decreased TNF Levels and Improved Retinal Ganglion Cell Survival in MMP-2 Null Mice Suggest a Role for MMP-2 as TNF Sheddase. Mediators Inflamm.

[54] Zhang X, Cheng M, Chintala SK (2004). Optic nerve ligation leads to astrocyte-associated matrix metalloproteinase-9 induction in the mouse retina. Neurosci Lett.

[55] Halfter W, Willem M, Mayer U (2005). Basement Membrane-Dependent Survival of Retinal Ganglion Cells. Invest Ophthalmol Vis Sci.

[56] Osenkowski P, Toth M, Fridman R (2004). Processing, shedding, and endocytosis of membrane type 1-matrix metalloproteinase (MT1-MMP). J Cell Physiol.

[57] Gariano RF, Hu D, Helms J (2006). Expression of angiogenesis-related genes during retinal development. Gene Expr Patterns.

[58] Hofmaier F, Hauck SM, Amann B, Degroote RL, Deeg CA (2011). Changes in matrix metalloproteinase network in spontaneous autoimmune uveitis model. Invest Ophthalmol Vis Sci.

[59] Smine A, Plantner JJ (1997). Membrane type-1 matrix metalloproteinase in human ocular tissues. Curr Eye Res.

[60] Takano A, Hirata A, Inomata Y, Kawaji T, Nakagawa K, Nagata S (2005). Intravitreal plasmin injection activates endogenous matrix metalloproteinase-2 in rabbit and human vitreous. Am J Ophthalmol.

